# Enhanced CT‑based radiomics model to predict CD40LG expression and clinical prognosis in head and neck squamous cell carcinoma

**DOI:** 10.1016/j.bjorl.2026.101820

**Published:** 2026-04-14

**Authors:** Liyuan Fan, Liang Shi, Haokun Yuan, Zhongchao Wang, Jinghan Wang, Feng Xian, Yandong Mu

**Affiliations:** a University of Electronic Science and Technology of China, School of Medicine, Sichuan Provincial People's Hospital, Department of Stomatology, Chengdu, China; b Southwest Medical University, The Affiliated Stomatology Hospital, Department of Prosthodontics, Luzhou, Sichuan, China; c Southwest Medical University, Institute of Stomatology, Luzhou, Sichuan, China; d Southwest Medical University, The Affiliated Hospital, Department of Pediatrics, Division of Neonatology, Luzhou, Sichuan, China; e Southwest Medical University, The Affiliated Stomatology Hospital, Department of Periodontics & Oral Mucosal Diseases, Luzhou, Sichuan, China; f North Sichuan Medical College, The Second Clinical Medical College, Nanchong Central Hospital, Department of Oncology, Nanchong, China

**Keywords:** CT, Radiomics, HNSCC, CD40LG, Clinical prognosis

## Abstract

**Objective:**

This research aimed to develop an enhanced CT-based radiomics model capable of predicting CD40LG expression.

**Methods:**

For 399 HNSCC cases, the Cancer Genome Atlas provided gene sequencing, and the Cancer Imaging Archive provided computerized tomography scans. Next, radiomic features were selected through maximum relevance minimum redundancy, and recursive feature elimination. Researchers used gradient boosting machines to develop a radiomics model for CD40LG prediction, evaluating it with operating characteristic curves, decision curves, and the Hosmer-Lemeshow test.

**Results:**

The study found that CD40LG levels were significantly lower in HNSCC tissues than healthy ones. High CD40LG expression was associated with better survival rates, as shown by Kaplan-Meier analysis. The findings of regression analyses confirmed that HNSCC patients with higher CD40LG expression had better outcomes. We developed a CD40LG prediction model based on seven key radiomics features. The ROC analysis showed high accuracy for our model, with an AUC of 0.872 for training data and 0.762 for validation data. The calibration and decision curves indicated good model performance in predicting outcomes and applicability. High CD40LG expression was linked to higher RS values, while HNSCC patients with lower RS values fared poorly.

**Conclusions:**

The study concludes that a contrast-enhanced CT-based radiomics model can accurately predict CD40LG expression and prognosis in HNSCC patients, aiding in clinical decision-making.

**Level of evidence:**

Level 4.

## Highlights

• CD40LG expression demonstrates independent prognostic significance in HNSCCs.
• A 7-feature CT radiomics model non-invasively predicts CD40LG expression.
• RS mapping CD40LG expression serves as an independent prognostic marker for HNSCCs.

## Introduction

Head and Neck Squamous Cell Carcinomas (HNSCCs) makeup 90% of head and neck cancers,^1^ with high mortality due to metastases and treatment resistance.^2^ Diagnosis involves costly methods like blood tests and biopsies,^3^^,^^4^ which may not accurately reflect tumors.^5^ Treatment mainly includes resection, radiation, and systemic therapy, with histopathology, Human Papillomavirus (HPV), and immunohistochemistry as common prognostic indicators.^6^, ^7^, ^8^, ^9^ However, histopathology samples are limited, and HPV testing varies by lab, highlighting the need for new prognostic indicators and personalized treatment strategies.^5^^,^^10^

CD40 Ligand (CD40LG) (also known as CD154), expressed in activated CD4+ T-cells,^11^ is crucial for inflammation and immune response,^12^ with its disorder linked to immune pathologies like atherosclerosis. CD40, its receptor, facilitates communication between innate and adaptive immunity and is part of key immune checkpoints.^13^, ^14^, ^15^ The CD40-CD40LG interaction activates immune cells, potentially leading to tumor cell death. In advanced HNSCC, reduced CD40 on antigen-presenting cells and decreased CD40LG on T-cells indicate impaired immunity.^16^ Pre-clinical studies show CD40 agonists can hinder tumor growth and improve prognosis.^17^ Current detection methods for CD40 and CD40LG rely on invasive immunohistochemistry, highlighting the need for more efficient detection techniques.

Radiology exams are essential for clinical assessment, with PET/CT (Positron Emission Tomography/Computerized Tomography) vital for staging HNSCC.^18^ Despite advancements in X-Ray, CT, MRI (Magnetic Resonance Imaging), and ultrasound, these methods fall short for precision medicine. Recently, Artificial Intelligence (AI) has shown great potential in healthcare,^19^ leading to the emergence of radiomics, which converts medical images into high-throughput data.^20^ This noninvasive method can identify tumor texture traits and has been used to classify and diagnose HNSCC early, assess tumor heterogeneity, and analyze molecular processes, though no studies have yet predicted CD40LG expression levels in HNSCC patients using radiomics.^20^

Therefore, this work made a novel proposal for using enhanced CT-based radiomics to predict the CD40LG expression level in HNSCC. Meanwhile integrating bioinformatics analysis attempted to explore the correlation between the putative molecular mechanisms as well as the immune microenvironment underlying the expression of CD40LG.

## Methods

### Data and image sources

The Cancer Genome Atlas (TCGA) provided transcriptional sequencing data, while the Cancer Imaging Archive (TGIA) supplied CT images. 399 HNSCC samples from TCGA were used in the relationship analysis between CD40LG expression and prognosis of HNSCC. To create and validate the radiomics model, 126 patients with preoperative enhanced CT scans in TCIA and transcriptome sequencing data in TCGA were enrolled in the study. 3D Slicer was used for image reconstruction, registration, segmentation, visualization, and quantitative analysis, while handling a variety of medical imaging data, including MRI, CT, ultrasound, and other modalities.

Bioinformatics analysis, the inclusion criteria: (1) Samples diagnosed as HNSCC, (2) Primary tumor samples of initial diagnosis and treatment, (3) HNSCC diagnosed as a primary solid tumor with RNA sequence data. The exclusion criteria: (1) Non-primary HNSCC, without OS data, or with an OS less than one month; (2) Samples with unknown tumor grades.

Based on the CD40LG expression dataset, in combination with additional inclusion and exclusion criteria. The inclusion criteria are samples with enhanced CT arterial phase imaging; the exclusion criteria are unqualified or post-treatment CT scans.

### Survival analysis and enrichment analysis

Using the survminer package of R, we determined the cutoff value for CD40LG expression. The application of an online site (www.xiantaozi.com) was able to differentiate the expression levels of CD40LG between tumor and normal tissues. In TCGA, the RNA sequence data in the level 3 HTSeq-FPKM (Fragments Per Kilobase per Million) format was uploaded and log2 transformed.

The survival times for high as well as low CD40LG expression groups were contrasted by the Kaplan-Meier (KM) curves. Univariate and multivariate Cox regression analyses were used to generate the characteristics' Hazard Ratios (HRs) and 95% present Confidence Intervals (CIs). Subgroup analysis and interaction testing were conducted afterward. Spearman's rank correlation coefficient was utilized to validate the relationship between CD40LG expression and HNSCC clinical characteristics.

We assessed the immune cell infiltration in every HNSCC sample after uploading the gene expression matrices to the CIBERSORTx (https://cibersortx.stanford.edu) databases. In order to investigate the correlation between CD40LG expression and immunocyte infiltration, the Wilcox test was carried out. Using the R-package “limma” the degree to which immune cells penetrated CD40LG high as well as low expression groups was ascertained.

Gene Set Enrichment Analysis (GSEA^22^ of the Kyoto Encyclopedia of Genes and Genomes (KEGG^23^ and Hallmark gene sets were performed to investigate the molecular mechanisms underlying the CD40LG expression differences. Additionally, statistics are considered significant at p under 0.05.

### Radiomics features extraction

To determine the volumes of interest, two radiologists who have worked for more than five years manually drew the whole tumor area using the 3D Slicer application (version 4.10.2).^24^ During delineation, we used both plain and enhanced CT scans to locate the lesion by adjusting window settings. We compared bilaterally for abnormal soft tissue masses and areas of abnormal enhancement to identify the tumor region. We used the R software to randomly split the samples into a training set as well as a validation set with an eight-to-two ratio. We also assessed how the two groups' intergroup variances differed.

The radiomics features’ consistency was judged by the Intraclass Correlation Coefficient (ICC).

30 samples were chosen at random through the stochastic number table approach after all of the cases had been sketched by one doctor. These samples were then examined and verified by the second doctor. Features that met the criteria were further screened.

### Radiomics features selection by mRMR and RFE

The mRMR^25^ filter not only considers the correlation between features and CD40LG but also considers the correlation between features. The RFE algorithm^26^ eliminated the less-important features in turn. Firstly, the ICC was employed to choose the features. Then, through the mRMR method, the top 20 features, further the optimal features subset, were screened by the RFE algorithm.

### Radiomics model establishment

The Gradient Boosting Machine (GBM) algorithm,^27^ which uses a set of weak classifiers (usually a decision tree), trains the newly added weak classifiers based on the negative gradient information of the current model's loss function and then combines the trained weak classifiers into the existing model in a cumulative form to construct a predictive model. Modeling of screened radiomics features by gradient enhancement model for prediction of CD4OLG expression.

### Evaluation of radiomics model

To validate the model's capability, a lot of indices were included, such as Accuracy (ACC), Specificity (SPE), Sensitivity (SEN), Positive Predictive Value (PPV), and Negative Predictive Value (NPV). ROC curves provide a comprehensive view of model performance. The calibration level of this radiomics model was determined by the calibration curve and the Hosmer-Lemeshow goodness-of-fit testing method. The Brier Score was utilized to judge the general capability of this prediction model. The decision curve displayed the clinical benefit. In a bid to contrast the AUC values of the training as well as validation sets and assess the fitting situation, the DeLong test was applied.

### Prognostic significance of the radiomics model

Using the probability cutoff value established by the survminer R program, the samples were categorized into RS high as well as low groups. To show the variations in OS within these two groups, KM curves were constructed. The Cox regression was carried out to conduct survival analysis and then subgroup analysis as well as interaction test.

### Statistical analysis

For all statistical calculations, R software (version 4.1.0) was utilized. It was deemed significant in statistics as long as p-values were below 0.05. To compare the baseline characteristics of the categorical variables, the Chi-Square test was utilized. The function of these indicators, such as ACC, SPE, SEN, PPV, and NPV was to measure the radiomics model's capacity.

## Results

### Patient stratification and clinical characteristics

A critical value of 0.2081 was used to divide the 399 HNSCC patients from the TCGA database into the CD40LG high-expression (200 samples) and low-expression (199 samples) groups for survival analysis. Table 1 embodied a discrepancy in the clinical characteristics. Comparing of the CD40LG high and low expression groups, there were significant statistics variations in the distribution of pathologic stage (p-equal 0.037), primary tumor (p-equal 0.002), and perineural invasion (p-equal 0.02).

**Table 1:** Clinical features of different CD40LG expression groups in HNSCC patients.

Variables	Total (n = 399)	Low (n = 199)	High (n = 200)	p
Age, n (%)				0.207
<60	165 (41)	89 (45)	76 (38)	
≥60	234 (59)	110 (55)	124 (62)	
Margin_status, n (%)				0.102
Negative	276 (69)	142 (71)	134 (67)	
Close	35 (9)	20(10)	15(8)	
Positive	45 (11)	23(12)	22(11)	
Unknown	43(11)	14(7)	29(14)	
Pathologic_stage, n (%)				0.037
I/II	78(20)	35(18)	43(22)	
III/IV	264(66)	143(72)	121(60)	
Unknown	57(14)	21(11)	36(18)	
Histologic_grade, n (%)				0.379
G1/G2	294(74)	151(76)	143(72)	
G3/G4	105(26)	48(24)	57(28)	
Primary_tumor_site, n (%)				0.002
Larynx	91 (23)	41 (21)	50 (25)	
Oral Cavity	255 (64)	142 (71)	113 (56)	
Oropharynx/Hypopharynx	53 (13)	16 (8)	37 (18)	
Gender, n (%)				0.93
Female	112 (28)	55 (28)	57 (28)	
Male	287 (72)	144 (72)	143 (72)	
Perineural_invasion, n (%)				0.02
NO	44 (36)	67 (34)	77 (38)	
Unknown	122 (31)	53 (27)	69 (34)	
YES	133 (33)	79 (40)	54 (27)	
Systematic_therapy, n (%)				0.05
NO	270 (68)	144 (72)	126 (63)	
YES	129 (32)	55 (28)	74 (37)	
Radiotherapy, n (%)				0.45
NO	190 (48)	99 (50)	91 (46)	
YES	209 (52)	100 (50)	91 (46)	

### Expression levels and survival analysis

**Fig. 1:**
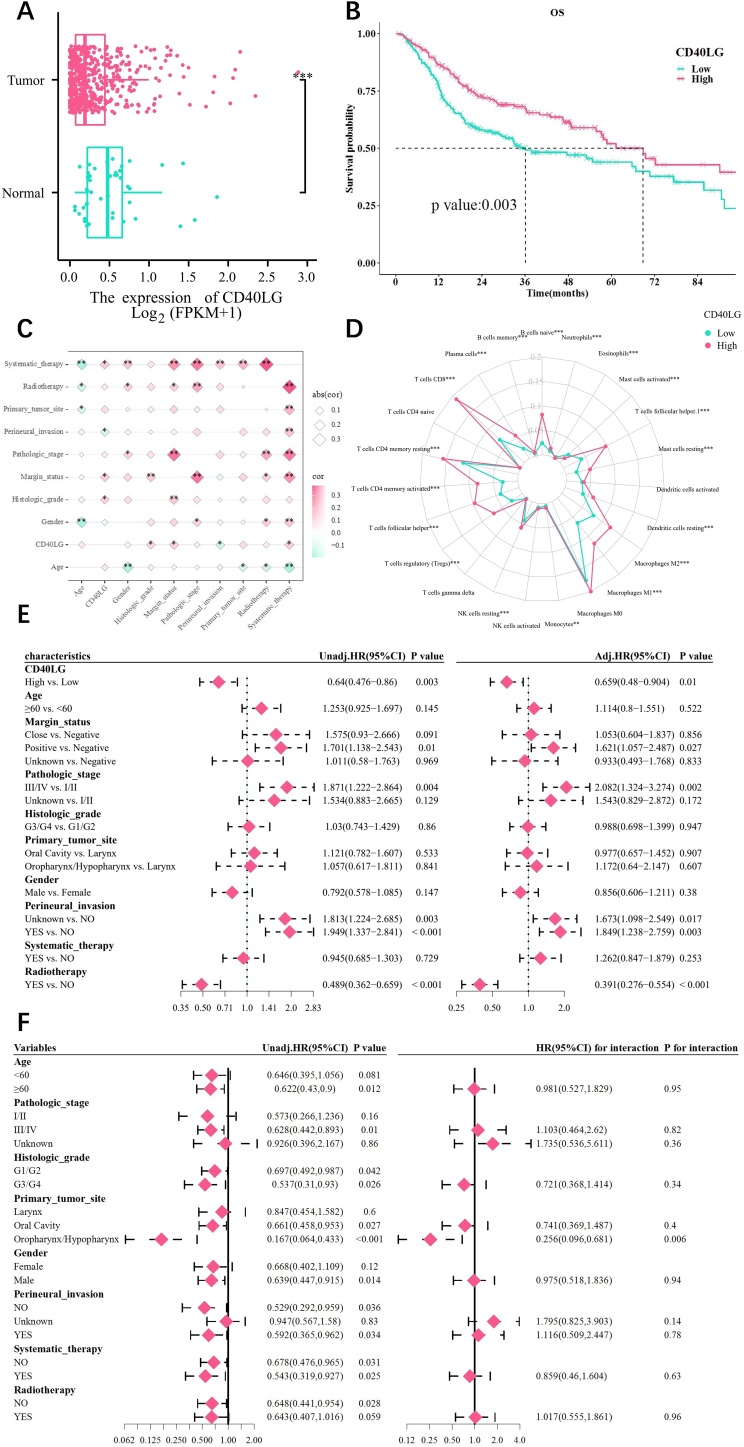
Correlation between CD40LG and clinical features. (A) Expression of CD40LG in normal as well as tumor tissues. (B) Correlation between CD40LG expression level and OS. (C) The correlation between CD40LG expression and HNSCC clinical features. (D) CD40LG expression as well as distribution in 23 different types of immune cells. (E) Univariate and Multivariate analysis. (F) Subgroup and interaction analysis. (no significance, p ≥ 0.05; * p < 0.05; ** p < 0.01, *** p < 0.001.).

As seen in Fig. 1A, HNSCC tissues had considerably lower levels of CD40LG expression than normal tissues (p-under 0.001). Aside from that, the median survival times were 36.03-months (95% CI 26.8–70.66) and 68.8-months (95% CI 55.7–110.46) in the CD40LG low as well as high expression groups, respectively. According to KM curve analysis, higher levels of CD40LG expression were substantially linked with OS improvement (p-equal 0.003) (Fig. 1B).

In Fig. 1E, CD40LG high expression was a protective factor for OS in the univariate Cox regression analysis (HR-equal 0.64; 95% CI 0.476 to 0.86; p-equal 0.003). As shown by the findings of multivariate Cox regression analysis, the high level of CD40LG expression continued to be a protective prognostic indicator for OS (HR-equal 0.659; 95% CI 0.48 to 0.904, p-equal 0.01). As illustrated in Figure 1 F, the analysis of the primary-tumor-site group demonstrated that in oropharynx/hypopharynx subfraction, the addition of CD40LG could prolong life survival. With a p-value of 0.006, the interaction test revealed a significant relationship for OS between CD40LG and the oropharynx/hypopharynx in the primary tumor site subgroup.

### Correlation with clinical outcomes and immune cell infiltration

It was shown in Fig. 1C CD40LG expression that a strong positive relationship with the Histologic grade, Margin status, and Systematic therapy. Conversely, the expression of CD40LG was inversely associated with perineural invasion (all p-under 0.05). The outcome of immunocyte infiltration suggested that the CD40LG high expression group had considerably greater infiltration of CD8 T-cells and activated memory CD4 T-cells (p-under 0.001), whereas Macrophages (M0 cell) had no significant differences in infiltration within the high and low expression of CD40LG groups (p-more than 0.05) (Fig. 1D).

**Fig. 2:**
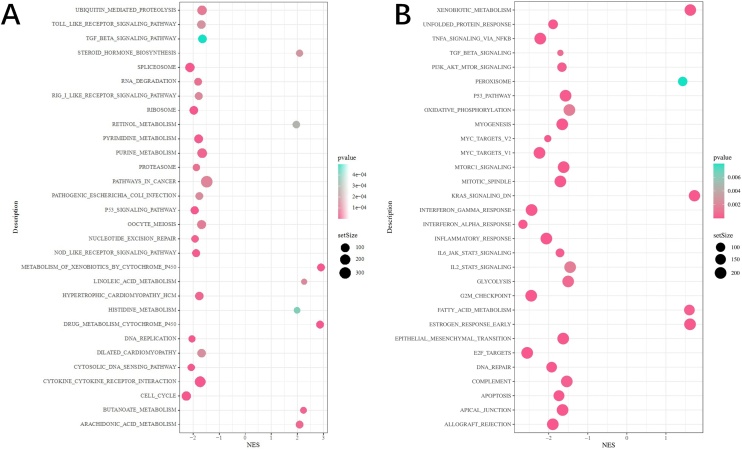
Analysis of the DEGs related to CD40LG expression. (A) KEGG enrichment analysis. (B) Hallmark enrichment analysis.

On the basis of KEGG enrichment analysis, the Differential Expressed Genes (DEGs) were significantly enriched in the p53 signal pathway and cell cycle-related pathways in the group showing low expression of CD40LG (Fig. 2A). In the Hallmark gene set, the DEGs were significantly abundant in p53 and G2M checkpoint signaling pathways (Fig. 2B).

### Radiomics model construction and evaluation

The samples were randomly grouped into 102 samples for the training set and 24 for the validation set. Between these two groups, the baseline status of the samples was consistent and comparable (Table 2).

**Table 2:** Compare the discrepancies between the training group and the validation group.

Variables	Total (n = 126)	Train (n = 102)	Validation (n = 24)	P
CD40LG, n (%)				1
Low	58 (46)	47 (46)	11 (46)	
High	68 (54)	55 (54)	13 (54)	
Age, n (%)				0.871
< 60	57 (45)	47 (46)	10 (42)	
≥ 60	69 (55)	55 (54)	14 (58)	
Margin_status, n (%)				0.401
Negative	88 (70)	70 (69)	18 (75)	
Close	9 (7)	7 (7)	2 (8)	
Positive	11 (9)	11 (11)	0 (0)	
Unknown	18 (14)	14 (14)	4 (17)	
Pathologic_stage, n (%)				0.544
I/II	24 (19)	18 (18)	6 (25)	
III/IV	80 (63)	67 (66)	13 (54)	
Unknown	22 (17)	17 (17)	5 (21)	
Histologic_grade, n (%)				0.715
G1/G2	88 (70)	70 (69)	18 (75)	
G3/G4	38 (30)	32 (31)	6 (25)	
Primary_tumor_site, n (%)				0.471
Larynx	31 (25)	27 (26)	4 (17)	
Oral Cavity	76 (60)	61 (60)	15 (62)	
Oropharynx/Hypopharynx	19 (15)	14 (14)	5 (21)	
Gender, n (%)				0.99
Female	34 (27)	27 (26)	7 (29)	
Male	92 (73)	75 (74)	17 (71)	
Perineural_invasion, n (%)				0.913
NO	43 (34)	34 (33)	9 (38)	
Unknown	46 (37)	38 (37)	8 (33)	
YES	37 (29)	30 (29)	7 (29)	
Systematic_therapy, n (%)				1
NO	88 (70)	71 (70)	17 (71)	
YES	38 (30)	31 (30)	7 (29)	
Radiotherapy, n (%)				1
NO	57 (45)	46 (45)	11 (46)	
YES	69 (55)	56 (55)	13 (54)	
OS, n (%)				0.882
Alive	83 (66)	68 (67)	15 (62)	
Dead	43 (34)	34 (33)	9 (38)	
OS. time, Median (Q1, Q3)	29.75 (14.05, 49.97)	27.03 (13.61, 50.25)	33.17 (14.95, 46.17)	0.968

ICC analysis showed that 100 (93.5%) features had an interobserver ICC value of ≥ 0.75, which was enrolled, while 7 (6.5%) features had an ICC value between 0.5 and 0.74, and 0 features had an ICC value under 0.5. Furthermore, a total of 107 extracted radiomics features with an ICC value of not less than 0.75 met the requirements for further analysis.

**Fig. 3:**
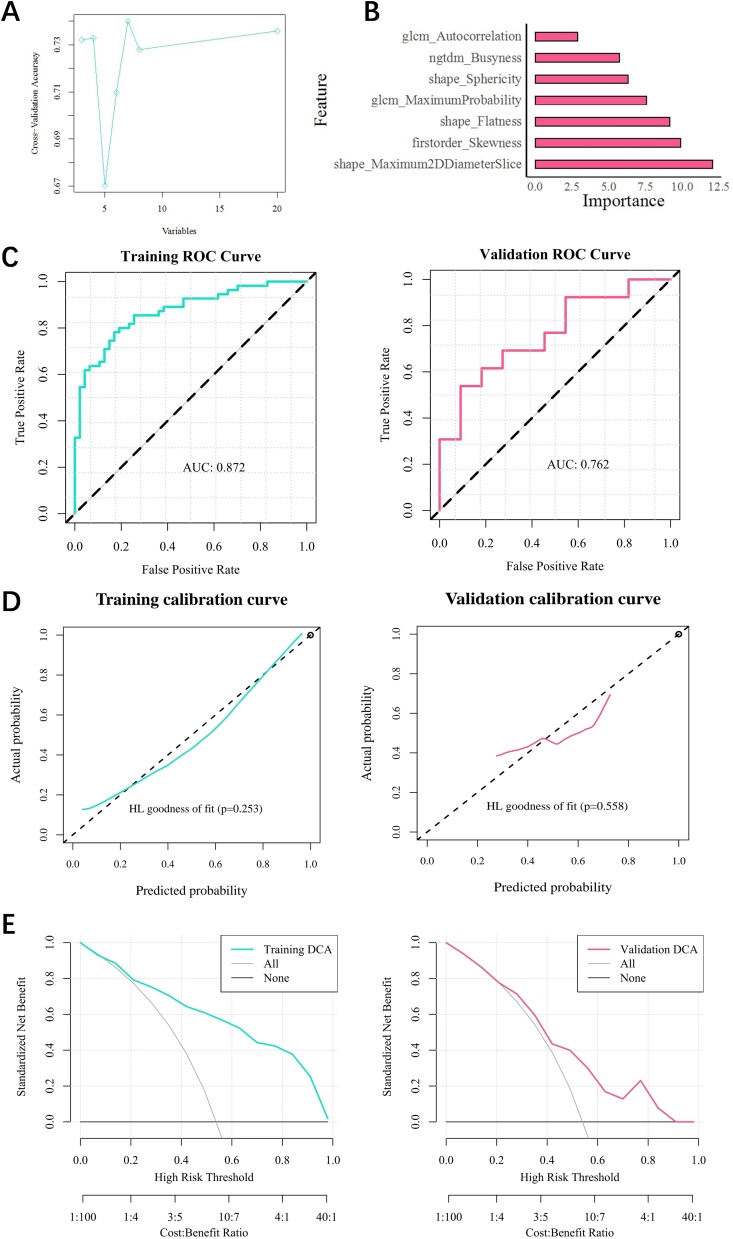
Radiomics model’s construction and evaluation. (A) Radiomics features selection. (B) The importance of radiomics features in GBM algorithm. (C) ROC curve analysis. (D) Calibration curve analysis. (E) Hosmer-Lemeshow test (no significance, p ≥ 0.05; * p < 0.05; ** p < 0.01, *** p < 0.001.).

The top 20 features were selected using the mRMR method from ICC analysis. Furthermore, RFE was employed to screen out the optimal seven features, which include glcm Autocorrelation, ngtdm Busyness, shape Sphericity, glcm Maximum Probability, shape Flatness, first order Skewness and shape Maximum2DdiameterSlice (Fig. 3A). In Figure 3B, the importance of features was displayed.

We developed a radiomics model with good predictive performance. The ROC curves’ AUC values were 0.872 in the training set and 0.762 in the validation set (Fig. 3C). According to the calibration curve analysis and Hosmer-Lemeshow test, the radiomics model's ability to predict the expression level of CD40LG conforms to the actual value (p-more than 0.05) (Fig. 3D). Additionally, decision curves showed that the model was more clinically applicable (Fig. 3E).

### Prognostic significance of the radiomics model

The prognostic significance analysis was then performed on 126 HNSCC patients simultaneously obtained from the TCGA and TCIA. The cutoff value for RS, which was 0.563, was employed to classify the samples into two groups: RS high (63) and RS low (63) groups. In these two groups, the age subgroup did not vary significantly (p-equal 0.283). However, the distribution of the pathologic stage and primary tumor site differed significantly (p-equal 0.001 and p-equal 0.016, respectively).

**Fig. 4:**
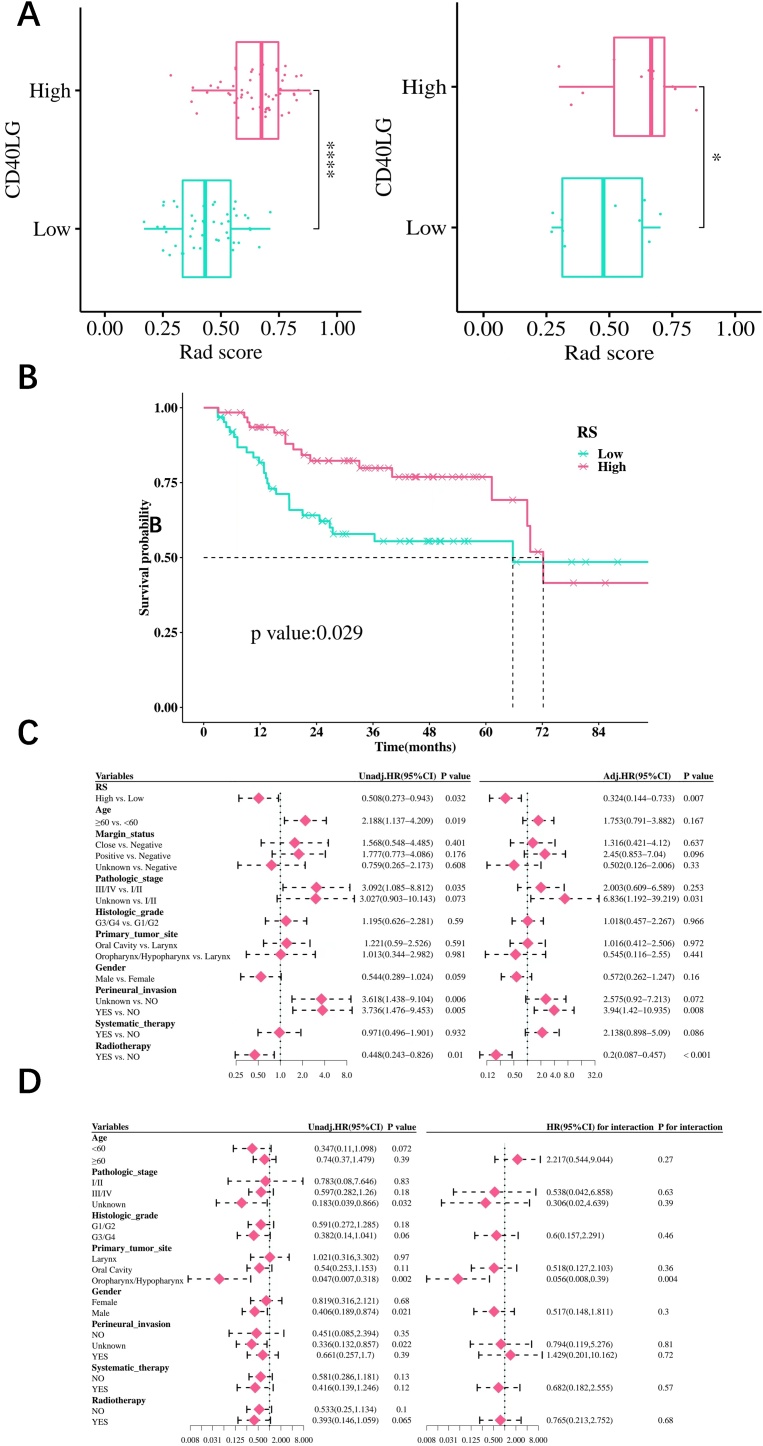
The correlation between RS and clinical prognosis. (A) Comparing the differences in RS between the degree of CD4OLG expression. (B) The relationship between RS and OS. (C) Univariate and Multivariate Cox regression analysis. (D**)** Subgroup analysis and interaction test. (no significance, p ≥ 0.05; * p < 0.05; ** p < 0.01, *** p < 0.001).

In the training as well as validation sets, the CD40LG high-expression group's RS value was found to be high (p-under 0.05) (Fig. 4A). The survival analysis for the two groups is presented in Fig. 4B. According to the KM curves, compared to the RS high group, the RS low group's OS was worse (p-equal 0.029). The high RS was found to be a protective factor for HNSCC patients (p-under 0.05) (Fig. 4C). The result of univariate subgroup analysis indicated that there were significant interactive effects among RS and the oropharynx/hypopharynx primary tumor site subgroup (p-equal 0.04) (Fig. 4D).

## Discussion

HNSCC, with nearly 350,000 annual deaths, is aggressive and heterogeneous, making the identification of novel prognostic markers crucial.^28^^,^^29^ Our study found a significant link between CD40LG and HNSCC prognosis, leading to the development of a radiomics model that effectively predicts CD40LG expression levels. High RS from this model correlates significantly with im- proved OS in HNSCC patients. As is known to all, CD40 and CD40LG are the important targets for tumor immunotherapy. Several cancers may be directly inhibited by the activation of CD40.^30^ According to research by Nicholas F. Kuhn et al., CD40LG-modified chimeric antigen receptor T-cells improve anticancer activity by preventing tumor immune escape.^31^ However, the detection methods of CD40LG used in clinical are limited. Radiomics, combining imaging and machine learning, is gaining academic interest. Our study shows CD40LG's reliability as a prognostic indicator in HNSCC patients, with high expression linked to improved Overall Survival (OS). Cox regression analyses confirm CD40LG overexpression as an independent protective factor for OS, suggesting a radiomics model could aid in predicting CD40LG expression and enhance precision medicine in HNSCC. Notably, in our multivariate Cox regression analysis, the radiomics score remained an independent protective factor for overall survival after adjusting for clinical-pathological characteristics including TNM stage and histologic grade (HR = 0.324, 95% CI 0.144‒0.733, p = 0.007). This indicates that RS provides additional prognostic value beyond conventional staging systems, highlighting its potential as a complementary tool for risk stratification in HNSCC patients.

Traditional imaging relies on morphology for diagnosis, lacking objective evaluation, while radiomics enables quantitative analysis and extracts hidden features from images.^21^ One-slice CT image-based kernelized radiomics model was given by Junyong Ye et al., and this model accurately predicted HNSCC grades using a self-created one-slice dataset.^32^ The model yielded an AUC of 95.91%. Another study by Tang et al. demonstrated that radiomics features of the planned target volume and gross tumor volume are accurate prognosis and recurrence forecasting tools on HNSCC patients, which may assist doctors in developing individualized treatment plans.^33^ Zhu et al. reviewed and analyzed the data of 126 HNSCC patients in TCGA and TCIA databases. The relationship between radiomics and genomic characteristics was assessed by gene enrichment analysis and linear regression. Additionally, the random-forest classifier was utilized to foresee the HPV and TP53 gene states based on radiomics traits. The anticipated AUC were 0.71 and 0.641, respectively.^34^ In the present study, the radiomics model for estimating the HNSCC patients’ prognosis was constructed using the GBM algorithm in conjunction with the mRMR and RFE feature selection methods. The value of AUC is higher, which indicates better predictive performance. Thus, the prediction efficacy across the training and validation teams was outstanding and steady, with AUCs of 0.872 and 0.762, respectively.

Previous studies have demonstrated that CD40LG holds significant clinical relevance in Head and Neck Squamous Cell Carcinoma (HNSCC): expression of multiple immune checkpoint ligands correlates with prognosis, with CD40 L expression associated with markedly improved survival outcomes, suggesting its potential as a favorable immunological prognostic marker^35^ Concurrently, CD40LG has been identified as one of the most significant independent prognostic factors, substantially enhancing the predictive efficiency of prognostic models.^36^ The RS derived from our model can serve as a non-invasive surrogate for CD40LG expression. Compared to traditional molecular testing that requires invasive tissue samples, RS is more accessible and can be easily obtained from routine enhanced CT scans. This facilitates risk stratification and personalized treatment planning for HNSCC patients. Moreover, RS can be used for dynamic monitoring during follow-up, aiding in treatment response assessment and disease management. This non-invasive approach has the potential to directly inform clinical decision-making, particularly in resource-limited settings. Zhao et al. confirmed that CT-based radiomics may enhance preoperative nodal status identification. It could specify patient groups that would most benefit from neck therapy and support clinical judgment.^37^ Another study further indicated that in comparison to a simple clinical characteristic model, a CT-based radiomics model has comparable or even greater advantages in predicting the prognosis of HNSCC.^38^

Radiomics has demonstrated significant promise, but there are still several challenges. Firstly, all materials were retrieved from publicly available datasets (TCGA/TCIA), which have a wide range of image quality and may not represent global patient populations. This limits the generalizability of our findings. External validation in multi-center, prospective cohorts is needed to confirm robustness and translatability. Secondly, radiomics relies on high-quality image data and accurate segmentation, which can be labor-intensive.^39^ Thirdly, clinical practice lacks guidance from multicenter prospective radiomics research. Furthermore, radiomics features are highly sensitive to variations in imaging protocols, scanner manufacturers, and reconstruction algorithms, which may introduce technical heterogeneity and compromise reproducibility.^40^^,^^41^ The “small-n, large-p” problem ‒ where the number of features far exceeds the sample size ‒ also raises concerns about overfitting and model robustness.^42^ Additionally, the biological interpretability of radiomics remains limited; while our model correlates with CD40LG expression, it does not directly measure biological function and may be influenced by confounding factors such as tumor microenvironment heterogeneity or differences in information scales between imaging and genomic data. Additionally, our study did not systematically account for tumor characteristics such as HPV status, which are known to influence biological behavior and may confound radiomic feature extraction.^15^ Despite these limitations, if validated, our model could contribute to global cancer burden reduction by providing a non-invasive tool for CD40LG prediction, especially in resource-limited settings where tissue sampling is challenging.

Furthermore, it is crucial to emphasize that the Radiomics Score (RS) derived from our model should not be interpreted as a standalone biomarker to guide therapeutic decisions, particularly regarding immunotherapy targeting the CD40/CD40LG pathway. While a high RS is associated with favorable prognosis and may reflect a potentially more immunoreactive tumor microenvironment, it does not directly measure the functional state of the CD40/CD40LG axis or the suitability of a patient for specific immunotherapies. The decision to initiate any targeted therapy must rely on robust molecular profiling and validated clinical biomarkers, not on radiomics signatures alone. Therefore, the clinical utility of our model at its current stage is exploratory; it serves as a non-invasive hypothesis-generating tool that could complement, but never replace, standard pathological and molecular assessments for patient stratification in future research.

## Conclusion

Our findings demonstrated that the prognosis of HNSCC might be greatly influenced by the expression levels of CD40LG. We can accurately forecast the expression levels of CD40LG using a contrast-enhanced CT radiomics model. As a non-invasive and promising tool for predicting tumor prognosis, this model has the potential to be extensively adopted in clinical practice.

## ORCID ID

Liang Shi: 0009-0009-6058-4785

Haokun Yuan: 0000-0001-8614-7756

Zhongchao Wang: 0000-0002-9460-0255

Jinghan Wang: 0009-0008-3821-8909

Feng Xian: 0000-0002-1310-3506

Yangdong Mu: 0000-0002-1829-6116

## Funding

This work was supported by the Chengdu Science and Technology Bureau Project (Grant nº 2024-YF09-00026-SN).

## Meeting of ethical standards

This study used only publicly available, anonymized datasets (TCIA/TCGA) and did not involve any interaction with patients or access to personal identifying information. As such, institutional ethical approval was not required. All data were collected in compliance with relevant data protection and privacy regulations.

## Data availability statement

The authors declare that all data are available in repository.

## Declaration of competing interest

The authors declare no conflicts of interest.
